# Clinical utility of the Prostate Health Index (*phi*) for biopsy decision management in a large group urology practice setting

**DOI:** 10.1038/s41391-017-0008-7

**Published:** 2017-11-20

**Authors:** Jay White, B. Vittal Shenoy, Ronald F. Tutrone, Lawrence I. Karsh, Daniel R. Saltzstein, William J. Harmon, Dennis L. Broyles, Tamra E. Roddy, Lori R. Lofaro, Carly J. Paoli, Dwight Denham, Mark A. Reynolds

**Affiliations:** 1 Carolina Urology Partners, Huntersville, NC USA; 2 Chesapeake Urology Associates, Towson, MD USA; 3 The Urology Center of Colorado, Denver, CO USA; 4 Urology San Antonio, P.A., San Antonio, TX USA; 5 Beckman Coulter, Carlsbad, CA USA; 6 Beckman Coulter, Brea, CA USA

**Keywords:** Molecular neuroscience, Schizophrenia

## Abstract

**Background:**

Deciding when to biopsy a man with non-suspicious DRE findings and tPSA in the 4–10 ng/ml range can be challenging, because two-thirds of such biopsies are typically found to be benign. The Prostate Health Index (*phi*) exhibits significantly improved diagnostic accuracy for prostate cancer detection when compared to tPSA and %fPSA, however only one published study to date has investigated its impact on biopsy decisions in clinical practice.

**Methods:**

An IRB approved observational study was conducted at four large urology group practices using a physician reported two-part questionnaire. Physician recommendations were recorded before and after receiving the *phi* test result. A historical control group was queried from each site's electronic medical records for eligible men who were seen by the same participating urologists prior to the implementation of the *phi* test in their practice. 506 men receiving a *phi* test were prospectively enrolled and 683 men were identified for the historical control group (without *phi*). Biopsy and pathological findings were also recorded for both groups.

**Results:**

Men receiving a *phi* test showed a significant reduction in biopsy procedures performed when compared to the historical control group (36.4% vs. 60.3%, respectively, *P* < 0.0001). Based on questionnaire responses, the *phi* score impacted the physician’s patient management plan in 73% of cases, including biopsy deferrals when the *phi* score was low, and decisions to perform biopsies when the *phi* score indicated an intermediate or high probability of prostate cancer (*phi* ≥36).

**Conclusions:**

*phi* testing significantly impacted the physician’s biopsy decision for men with tPSA in the 4–10 ng/ml range and non-suspicious DRE findings. Appropriate utilization of *phi* resulted in a significant reduction in biopsy procedures performed compared to historical patients seen by the same participating urologists who would have met enrollment eligibility but did not receive a *phi* test.

## Introduction

Prostate-specific antigen (PSA) screening for prostate cancer (PCa) has come under increasing scrutiny in recent years. Despite a documented reduction in men presenting with metastatic disease since PSA was first introduced in the early 1990s [1, 2], its debatable impact on overall survival and an increasing concern about over-diagnosis of indolent cancer has led to restricted recommendations regarding its use [3–5]. The US Preventative Services Task Force (USPSTF) recommended against PSA screening for men of any age in 2012 [6], although an updated draft recommendation statement was recently issued for public comment, wherein the USPSTF acknowledged that PSA testing may be appropriate for certain men in the 55–69 year age range (C-recommendation) [7].

Another problem associated with PSA testing is its relatively poor diagnostic specificity. According to the National Comprehensive Cancer Network (NCCN), ~85% of men with PSA levels <4 ng/ml are found to have non-cancerous biopsy findings, whereas men with PSA levels between 4–10 ng/ml have ~30–35% chance of a positive biopsy result [8]. This potentially exposes over two-thirds of such men to complications associated with prostate biopsies such as bleeding, pain, and the risk of infection. Given these limitations, there is considerable interest in new biomarker panels demonstrating improved clinical specificity for PCa detection.

The Beckman Coulter Prostate Health Index (*phi*) combines the results of three quantitative kallikrein immunoassays, total PSA (tPSA), free PSA (fPSA), and [-2]proPSA (p2PSA) into a single numerical score (the *phi* score): (p2PSA/fPSA × √tPSA). It was approved by the Food and Drug Administration (FDA) in 2012 for use as an aid in distinguishing PCa from other benign prostatic conditions in men aged 50 years having non-suspicious digital rectal exam (DRE) findings and with serum tPSA levels ranging from 4 to 10 ng/ml [9]. The pivotal clinical trial submitted for FDA approval included 658 men who met the above criteria and ranged in age from 50 to 84 years. The *phi* test showed a significant improvement in PCa detection when compared with tPSA and %fPSA. For example, a *phi* score of 27.0 provided 31.1% clinical specificity at a sensitivity cutoff of 90%. This represented nearly a 3-fold improvement in PCa detection compared with tPSA testing alone.

An expanded version of the multicenter study described above was published, including 892 men with serum tPSA levels ranging from 2 to 10 ng/ml [10]. An increasing *phi* score was associated with a 4.7-fold increased risk of prostate cancer and a 1.61-fold increased risk of aggressive cancer (Gleason score ≥7) on biopsy. Additionally, the improved diagnostic performance of *phi* has been demonstrated in numerous other published clinical studies world-wide [11–26]. A meta-analysis from eight such studies [27], representing 2919 patients in total, showed a pooled clinical specificity of 31.6% at the 90% sensitivity threshold (95% Cl, 29.2%–34.0%).

Despite the proven diagnostic performance of *phi*, only one published study to date has evaluated its clinical utility in real world practice [28]. The purpose of the current study was to prospectively examine the impact of *phi* testing on biopsy decisions in four large urology group practices. The primary objective was to examine how *phi* influenced the physician’s management plan using a two-part questionnaire. The secondary objective was to compare biopsy procedures performed for patients receiving a *phi* test to historical controls seen by the same participating physicians before the *phi* test had been implemented in their practice.

## Materials and Methods

Sites were selected based on the commercial implementation of *phi* into their practice. Four large urology group practices agreed to participate in the study, representing geographically diverse regions across the United States. The protocol was approved by a central institutional review board (IRB) with waived consent because all patient information was de-identified. Men receiving a *phi* test result were prospectively enrolled from July 2015 through April 2017. Historical control patients were identified from each site within 24 months prior to initiating the study protocol.

### Study design

**Fig. 1:**
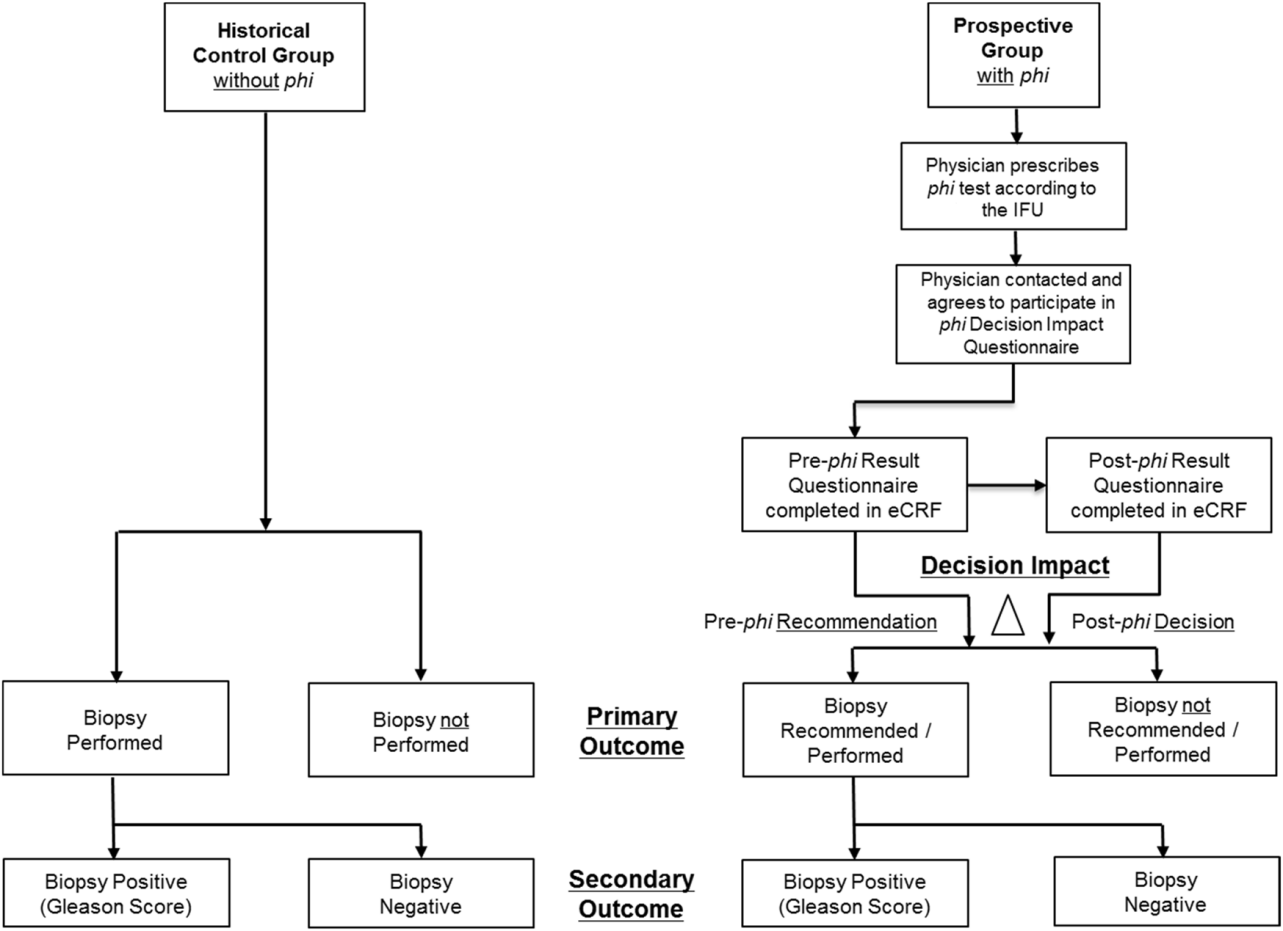
Diagram demonstrating patient’s flow through the study protocol

**Fig. 2:**
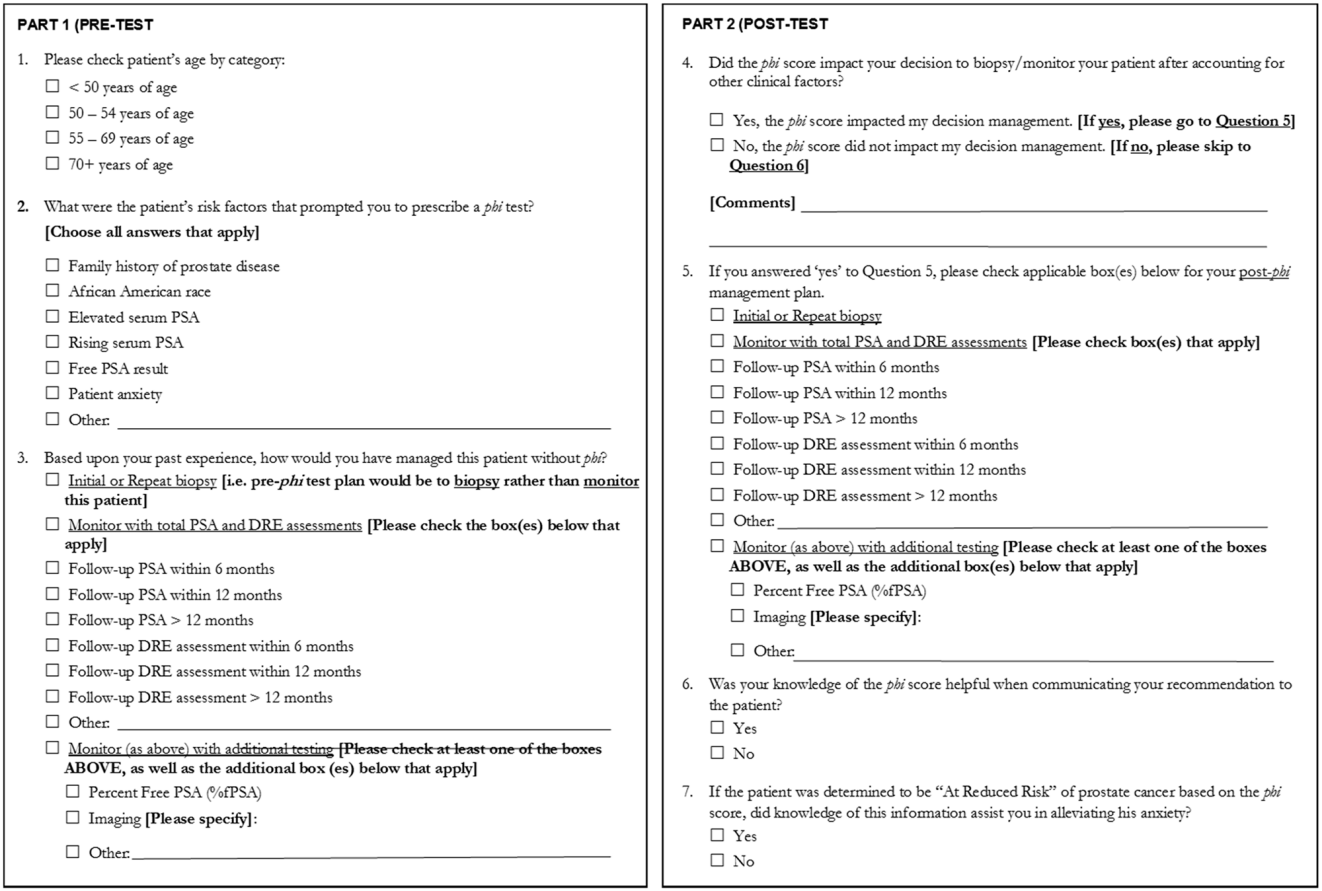
Physician Decision Impact Questionnaire

This was a prospective, observational study to determine if the use of *phi* testing changes physician behavior patterns when comparing their biopsy recommendations to a historical control group of similar patients seen by the same physicians (Fig. 1). A two-part questionnaire was used to document the physician’s preliminary patient management plan before receiving the *phi* result, compared to their final recommendation for that same patient after receiving the *phi* result (Fig. 2). Questions included whether or not knowledge of the *phi* result was helpful when communicating their final recommendation to patients. Clinical and pathological data for both patient groups was extracted from on-site electronic medical records.

### Subjects

Patients for whom a participating urologist requested a *phi* test were recruited at the time of blood draw for the prospective (observational) study group. Inclusion criteria: men ≥50 years of age with total serum PSA between 4–10 ng/ml and non-suspicious DRE findings. The prior PSA test was required to be performed within 6 months of the DRE. Exclusion criteria: men with a prior history of PCa, use of any dosage of 5-alpha reductase inhibitors within the previous 3 months, men with a previous biopsy result that was either positive or suspicious for prostate cancer (e.g., HGPIN, atypia), men with a history of prostatectomy for any reason, or men who had undergone transurethral resection of the prostate. Patients were also excluded if the physician decided not to wait for the *phi* result before performing a biopsy.

The historical cohort of patients was selected from each site’s electronic medical records for the purpose of establishing a baseline of practice patterns within the 24 months prior to their initiating the study protocol. Patients included in this group met the inclusion/exclusion criteria but did not receive a *phi* test as part of their assessment. For inclusion in the historical control group, patients had to be treated by the same physicians participating in the prospective arm of the study.

### Statistical analysis

The primary endpoint of *phi* impact on decision to biopsy was assessed for statistical significance using the Normal Approximation to the binomial test for proportions. Percentages for categorical responses from the two-part questionnaire were calculated from Total *N* or available data (where applicable). All data reporting and analyses were done using SAS Software version 9.4M3. All statistical tests were conducted with *ɑ* = 0.05, unless otherwise stated.

## Results

Our study comprised a total of 506 men in the prospective arm and 683 men in the historical control arm (Table 1). Patient age distributions, demographics, and clinical risk factors were balanced between the two arms.

**Table 1:** Patient characteristics

Characteristic		Prospective group (with phi) *n* = 506	Historical control group (without phi) *n* = 683
Age in years, *n* (%)	Mean ± SD	66.1 ± 7.1	65.8 ± 7.5
	50–54 years of age	27 (5.3)	57 (8.3)
	55–64 years of age	207 (40.9)	255 (37.3)
	65+ years of age	272 (53.8)	371 (54.3)
Most recent PSA score	Mean ± SD	5.9 ± 1.5	5.9 ± 1.5
	Median (range)	5.6 (4.0–10.0)	5.5 (4.0–10.0)
Race, *n* (%)	White	416 (82.2)	590 (86.4)
	Black	47 (9.3)	56 (8.2)
	Asian	5 (1.0)	5 (0.7)
	Native American	2 (0.4)	1 (0.1)
	Other	23 (4.5)	20 (2.9)
	Unknown	12 (2.4)	11 (1.6)
DRE results, *n* (%)	Non-suspicious	498 (98.4)	671 (98.2)
	Other*	8 (1.6)	12 (1.8)
Risk factors, *n* (%), not mutually exclusive	Family history of prostate disease	14 (9.0)	26 (17.0)
	African American race	21 (13.5)	8 (5.2)
	Elevated serum PSA	145 (93.5)	138 (90.2)
	Rising serum PSA	48 (31.0)	64 (41.8)
	%fPSA	9 (5.8)	13 (8.5)
	Other	0 (0)	8 (5.2)

*Other defined by physicians as a write-in which included enlarged (*n* = 15), R firmer (*n* = 1), benign (*n* = 1), nodule non (*n* = 1), uncertain (*n* = 1), and blank (*n* = 1)

Table 2 summarizes the frequency of biopsy procedures performed between the prospective and historical control groups. 36.4% of men in the prospective group received a biopsy (95% CI, 32.5%–40.9%), compared to the non-*phi* tested historical control group’s biopsy rate of 60.3% (95% CI, 56.3%–63.9%). This demonstrated a statistically significant reduction (*P* < 0.0001) in biopsy procedures for patients receiving a *phi* test result. In the prospective arm, 147 of 162 men (90.7%) were undergoing their first biopsy (22 of 184 men did not have a biopsy history reported), and in the historical arm, 339 of 355 men (95.5%) were undergoing their first biopsy (57 of 412 men did not have a biopsy history reported). The proportion of positive biopsy findings did not increase in the prospective group, while there was a modest decrease in the overall percentage of low-grade Gleason 6 tumors detected compared to the historical control group (9.9% vs. 18.4%, respectively).

**Table 2:** Biopsies performed and pathological findings by study group

Study group	Biopsies performed (% of total* n*)	Positive biopsies (% of Bx Perf.)	GS6 cancers detected (% of total *n*)
Prospective (with *phi*) *n *= 506	184 (36.4)	110 (59.8)	50 (9.9)
Historical control (without *phi*) *n *= 683	412 (60.3)	257 (62.4)	126 (18.4)

Table 3 summarizes the physician-reported decision impact of the *phi* test result on their patient management plan, based on their Pre-Test vs. Post-Test Questionnaire responses. Overall, 72.5% of the physician responses indicated that the *phi* score did impact their patient management plan after accounting for other clinical factors. This included 43% of cases where the physician reported a changed biopsy recommendation based on the *phi* result, and 19% of cases where the preliminary monitoring strategy was modified based on the *phi* result (i.e., more or less frequent follow-up and/or inclusion of additional testing such as magnetic resonance imaging (MRI)). Additionally, 92% of physician responses indicated that knowledge of the *phi* score was helpful when communicating their recommendation to the patient, including 28% of cases where a “reduced risk” *phi* score was helpful in alleviating the patient’s anxiety about the likelihood of significant cancer.

**Table 3:** Decision impact of *phi* on physician’s management plan based on Pre-Test vs. Post-Test Questionnaire Responses

					**Percent of total responses**
“Yes”, the *phi* score impacted my patient management plan after accounting for other clinical factors					72.5
Decided to monitor instead of biopsy base on “reduced risk” *phi* score					28.7
Decided to biopsy instead of monitor based on “elevated risk” *phi* score					14.3
Modified monitoring strategy based on *phi* score (frequency and type of testing)					18.9
Knowledge of *phi* score was helpful when communicating my recommendation to the patient					92.3
Knowledge of “reduced risk” *phi* score was helpful in alleviating the patient’s anxiety					28.3
***phi*** **score**	**Biopsy → Monitor**	**Monitor → Biopsy**	**Monitor → Monitor (modified strategy)**	**Biopsy → Biopsy**	**Monitor → Monitor (unchanged strategy)**
0–35.9	105	8	58	50	13
36–55+	39	64	37	123	5
Total	144	72	95	173	18

## Discussion

This large study represents the only multicenter study to date investigating the impact of *phi* testing on biopsy decisions for patients presenting with elevated serum PSA and non-suspicious DRE findings. Our study compared a prospective group of patients assessed with *phi* to a non-*phi* historical cohort evaluated by the same participating physicians within the previous 24 months. Physicians were more inclined to defer biopsy when *phi* testing was included in their overall assessment, resulting in a net 24% reduction in biopsies performed compared to the historical control group. In addition, we observed an overall reduction in the percentage of low-grade Gleason score 6 tumors detected with *phi*.

Most recently, Tosoian et al. [28] reported similar findings with *phi* testing in their large academic center practice at Johns Hopkins University. A prospective registry of 345 men receiving a *phi* result was compared to a contemporary cohort of 1318 men who did not undergo *phi* testing. Their comparative analysis showed that *phi* testing reduced the rate of biopsy procedures performed without changing the frequency of higher-grade cancers detected. Overall, 39% of men in their registry underwent a biopsy when *phi* was included in the assessment, representing a 9% reduction in the rate of prostate biopsy procedures performed compared to the control group (48%, *P* < 0.001). 91% of men with *phi* <27 who underwent a biopsy had either a non-cancerous result or low-grade (Gleason score 6) pathology, whereas 76% of men with *phi* >55 had Gleason score ≥7 cancers. For men receiving an MRI as part of their assessment, the *phi* score was also shown to provide complimentary information for ruling out significant prostate cancer.

The two-part questionnaire used in our study was helpful for elucidating how knowledge of the *phi* result impacted the physician’s management decision to perform a biopsy or monitor the patient. According to the responses, physicians were less inclined to biopsy patients receiving a low *phi* score, and more inclined to recommend biopsy for patients receiving an intermediate to high-risk *phi* score (*phi* ≥36). *phi* also improved the physician’s ability to communicate their recommendation to the patient, and helped alleviate patient anxiety in cases where the *phi* score was low.

Although our study was not sufficiently powered to demonstrate differences in pathological staging, a number of published studies have shown that elevated *phi* scores can predict higher-grade prostate cancers (Gleason score ≥7) [26, 29–36]. The predictive accuracy can vary depending on the patient cohort investigated, however. For example, a recently published review reported AUC values ranging from 0.707 to 0.82 [37]. The lowest AUC value excluded men with suspicious DRE findings [29], whereas the highest AUC value included 23.7% of men who reportedly had an abnormal DRE [34]. In all cases, *phi* predicted aggressive prostate cancer with AUC values that were significantly greater than those for %fPSA or tPSA.

As previously reported in simulated budget impact studies [38, 39], the addition of *phi* testing represents a cost-effective strategy for prostate cancer detection while avoiding unnecessary biopsies. The present study demonstrates this benefit in real world clinical practice.

Our study included four large urology group practices representing diverse regions across the United States. One strength of our study was the enrollment of patients seen by the same participating physicians for the prospective and historical control groups. Weaknesses include no use of randomization and lack of longitudinal follow-up. These were not utilized since our study was intended to be strictly observational and was designed to measure decision impact at the point of the urologist consultation. Further studies are needed to address questions of long-term patient outcomes from subsequent biopsy procedures and later episodes of care.

## Conclusions

This represents the largest prospective study to date investigating the clinical utility of *phi* testing for men undergoing a diagnostic assessment for PCa. Overall, *phi* impacted the physician’s patient management decision in 73% of observational cases. Fewer men were biopsied when *phi* testing was included in the assessment (36% vs. 60% historically), along with an overall reduction in the percentage of low-grade Gleason score 6 cancers detected. Our results show that appropriate utilization of *phi* can significantly modify physician behavior patterns and improve their ability to diagnose and manage their patients. We believe our study supports the routine use of *phi* testing for men presenting with elevated serum total PSA and non-suspicious DRE findings.
